# Does the landscape functionality approach provide insight into rangeland conditions in the Tanqua Karoo region, South Africa?

**DOI:** 10.7717/peerj.13305

**Published:** 2022-05-19

**Authors:** Manam Saaed, Shayne Jacobs, Mmoto Leonard Masubelele, Lesego Khomo, Igshaan Samuels

**Affiliations:** 1 Botany Department, Faculty of Arts and Sciences, University of Benghazi, Benghazi, Libya; 2 Conservation Ecology and Entomology Department, University of Stellenbosch, Stellenbosch, Western Cape, South Africa; 3 Conservation Services, South African National Parks, Pretoria, Gauteng, South Africa; 4 Environmental Sciences, University of South Africa, Johannesburg, Gauteng, South Africa; 5 Agricultural Research Council–Animal Production, University of the Western Cape, Bellville, Western Cape, South Africa; 6 Biodiversity and Conservation Biology Department, University of the Western Cape, Cape Town, South Africa

**Keywords:** Fetches, Landscape function analysis, Nutrient recycling potential, Patchiness, Tankwa Karoo National Park, Vegetation types

## Abstract

The harsh environmental conditions coupled with a long history of overgrazing have altered the ecology of the arid Tanqua Karoo rangelands in South Africa, which necessitates rehabilitation. However, a suitable method for monitoring rangeland function over time is required for sustainable management. In this study, vegetation characteristics and landscape function indices were used to rate and compare rangeland conditions in 43 sites distributed among three vegetation types: Tanqua Karoo, Tanqua Wash Riviere, and Tanqua Escarpment Shrubland, which occupy different landscapes in the Tankwa Karoo National Park. The results showed low values of vegetation volume (mean of 10.1 m^3^ per 100 m^−2^) and low vegetated patches (mean of 29% patches *vs* 71% fetches). The overall landscape function indices (soil stability, water infiltration, and nutrient recycling) were low and amounted to 55%, 28%, and 17%, respectively. Amongst the various examined landscapes, the escarpment had the highest values of most of the measured landscape functionality parameters, and the open plains had the lowest values. This revealed high heterogeneity of soil properties and vegetation characteristics amongst the different vegetation types, mainly influenced by altitudinal gradients. The higher-lying landscapes on the escarpment are relatively more functional and more susceptible to improvement when compared to the lower-lying landscapes on the plains. The landscape functionality approach (LFA) approach demonstrated that some of the examined vegetation types had insignificant improvement in landscape functionality likely not to improve in the near term due to existing low patchiness, higher fetch space and low LFA indices coupled with the low annual rainfall of the region. The landscape functionality approach has provided a suitable benchmark for assessing and monitoring the diverse vegetation types in this arid part of the world.

## Introduction

Arid rangeland conditions are influenced by a multitude of anthropogenic and environmental factors. Thus, it needs to be assessed and monitored continuously to inform the management and utilisation of the changes that occur over time. Information from monitoring is fundamental, especially when the rangelands are prone to be leaky and dysfunctional, particularly if the assessment results relate directly to management and decision-making implications at different levels (Tongway & Hindley, 2004a; Van der Merwe, Bezuidenhout & Bradshaw, 2015).

Although rangeland condition has traditionally been viewed in the context of resource availability for livestock, there has been an increased acceptance that functional integrity is a more appropriate way to view the health of rangelands (Le Maitre et al., 2007; Kwok, Eldridge & Oliver, 2011). Functional integrity has been defined in many ways, but broadly, it is the ability of landscapes to capture, retain, and use vital resources such as organic matter, seeds, nutrients, water, and soil efficiently (Ludwig et al., 2004). The Trigger-Transfer-Reserve-Pulse (TTRP) conceptual framework represents one model for understanding resource dynamics in a landscape and explains the degradation and improvement of the ecosystem as a continuous loss or gain of the vital resources (Ludwig et al., 2004; Le Maitre et al., 2007; Read et al., 2016).

The ability of landscapes to resist stress, *i.e*., stability or resistance, or recover from stress, *i.e*., resilience, is related to resource retention and production (Kwok, Eldridge & Oliver, 2011). The landscape may lose its inherent spatial heterogeneity or patchiness by over-exploitation of its natural resources. For example, overgrazing may reduce the vegetation cover and patchiness. Therefore, its ability to retain resources could be decline to create a dysfunctional state that might not be resilient and is unable to return to its previous, more functional state, even when the disturbance has been removed (Van der Walt et al., 2012).

Describing the impacts after degradation has already taken place in arid ecosystems is too late for ecosystem conservation. Natural resource management systems must include monitoring processes to ensure that resource reserves are not degrading (Noble & Brown, 1997), and indicators must be early warning signs (Ludwig & Freudenberger, 1997). Hence, landscape function analysis (LFA) over time is becoming increasingly crucial for ecosystem monitoring (Patil et al., 2001), particularly when assessing the efficiency of rehabilitation interventions in arid and semi-arid rangelands.

LFA is a method that assesses the functionality of landscapes. It is an in-the-field, indicator-based procedure that allows rapid assessment of how well a landscape functions as a biophysical system (Tongway, 2010). It was developed and successfully used by Tongway & Ludwig (1997a) to monitor the functional state of rehabilitating mine sites in the Australian rangelands. The use of the method has since expanded to monitor land conditions following different anthropogenic impacts throughout the world (Randall, 2004; Munro et al., 2012; Ludwig & Tongway, 1997).

Due to many environmental factors, especially the arid and semi-arid climate, rangelands are fragile ecosystems worldwide and prone to degradation. The concept and methodology of LFA’s are among the active techniques that have successfully assessed the degradation and monitoring of the rehabilitation process in rangeland ecosystems (Mahmoud et al., 2014; Munro et al., 2012; Patil et al., 2001; Tongway, 2010). LFA can be used to assess current rangeland condition, ecosystem improvement over time, and early detection of rehabilitation failure, hence allowing a change in remediation approach and techniques to evade or correct unsuitable techniques and methods during the rehabilitation process (Randall, 2004).

Across rangeland landscapes, resource transfers are strongly influenced by two primary landscape attributes: (1) terrain shape and slope and (2) patchiness, which is the dimensions and spacing of patches and fetches (inter-patches). These attributes are measured in the field along with gradient-oriented transects (Tongway & Ludwig, 1997b). These measurements of terrain and patch types and dimensions are reliable indicators of the landscape’s capacity to function in capturing and concentrating scarce resources (Tongway & Ludwig, 1997a), thus evaluating the landscape functionality. The patch areas, where resources tend to accumulate, provide more favourable habitats for vegetation and fauna when compared to the interpatch ‘fetch’ areas (Bastin et al., 2002; Kwok, Eldridge & Oliver, 2011). The area occupied by vegetated patches compared with the size of fetches is becoming an accepted and useful standard to assess rangeland functionality in arid and semi-arid environments (Holm, Loneragan & Adams, 2002b; Vandenbruwaene et al., 2011).

In this study, LFA was conducted to assess rangeland conditions in the dry and sensitive Tankwa Karoo National Park (TKNP). The arid climate and associated environmental factors that prevail in the area are particularly harsh for vegetation growth and development. As a consequence of heavy grazing over hundreds of years, the area is perceived to be in a degraded state regarding soil erosion, changes in vegetation composition, and a decrease in plant productivity (Saaed Manam, 2018).

Previous studies in the area focused on the physical environment and major plant communities (Rubin, 1998; Beukes & Ellis, 2003; Van der Merwe, Van Rooyen & Van Rooyen, 2008; Agricultural Research Council - Institute for Soil, Climate and Water, 2012; Van der Merwe, Bezuidenhout & Bradshaw, 2015; Saaed Manam, 2018; Saaed et al., 2018). However, there is insufficient information about landscape functionality and rangeland condition, particularly in this part of the Tanqua Karoo region. This study is among only a few studies investigating LFA in the arid protected areas in the entire African continent.

The study first examined the patterns of landscape functionality across different vegetation types in the TKNP. Secondly, it attempted to set benchmarks against which further assessment of rangeland condition and rehabilitation monitoring in the Tanqua Karoo region and similar arid areas could be evaluated. As a starting point to this study, we accepted the following as empirical indices of landscape condition: (1) landscape heterogeneity through measuring the number, dimensions, and spacing of patches and fetches, (2) soil surface indices that evaluate soil stability, water infiltration capacity, and nutrient cycling potential, and (3) vegetation measurements assessing the number of plants per unit area, mean intervals between plants, and canopy volume per unit area (*sensu*Tongway & Hindley, 2004b).

## Materials and Methods

### Study area

**Figure 1:**
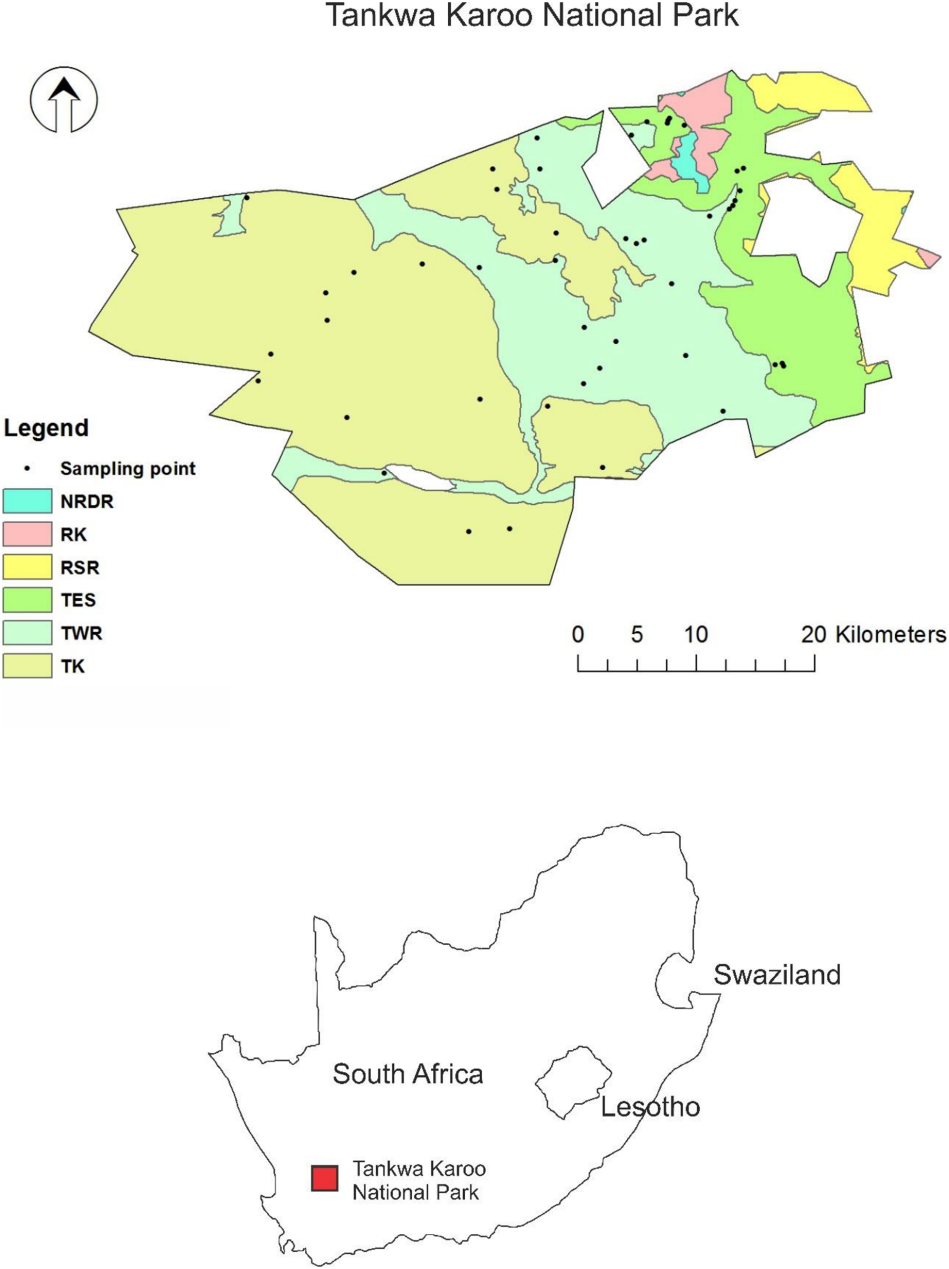
The geographical location of Tankwa Karoo National Park (TKNP) in South Africa. Sampling sites (dots) and different vegetation types occurring within the park boundaries. Vegetation types are: Nieuwoudtville-Roggeveld Dolerite Renosterveld (NRDR), Roggeveld Karoo (RK), Roggeveld Shale Renosterveld (RSR), Tanqua Escarpment Shrubland (TES), Tanqua Karoo (TK), and Tanqua Wash Riviere (TWR) (after Mucina & Rutherford, 2006). Focus in this paper is on TES, TK, and TWR.

Tankwa Karoo National Park, with an area of 1,486 km^2^, is an arid rangeland area in the northern section of the Tanqua Karoo region in South Africa (Fig 1). The rainfall is winter-dominant and ranges between 75 mm y^−1^ in the plains to 270 mm y^−1^ on the Roggeveld Mountains in the east (Saaed et al., 2018). The mean minimum temperature is −2.4 °C for July, and the mean maximum temperature is 36.6 °C for January.

The soils in the low-relief plains are shallow and, in some areas, including a desert pavement. In contrast, the soils on Roggeveld Escarpment are shallow stony lithosols (Francis et al., 2007). Shrubs and succulent plants are the main features of the area, with mass displays of flowering annuals occurring in spring after rainfall. Long-lived trees and sclerophyllous shrubs are rare and confined to watercourses (Saaed et al., 2018).

## Methods

The LFA methodology, as described by Ludwig et al. (1997) with a particular focus on soil surface condition assessment (Tongway & Hindley, 1995), was applied to the three main vegetation types in the park: the Tanqua Karoo (TK) which occupies the open plains, Tanqua Wash Riviere (TWR) which occupies the foot-slopes of the escarpment and floodplains, and Tanqua Escarpment Shrubland (TES) which occupies the Roggeveld Escarpment. The three vegetation types cover 93.3% of the Park’s surface area (TK = 53.9%, TWR = 26.5%, and TES = 12.9%). The fieldwork was conducted in February 2015. Using Arc GIS software and several environmental map layers, namely: NDVI from Landsat 8, vegetation, soil type, slope, habitat, and finally a cadastral layer, a total of 43 sampling sites were identified across the landscape to cover most areas of the chosen vegetation types and at different vegetation cover intensity. Of which 15 were within the TK, 15 were within the TWR, and 13 were within the TES.

**Figure 2:**
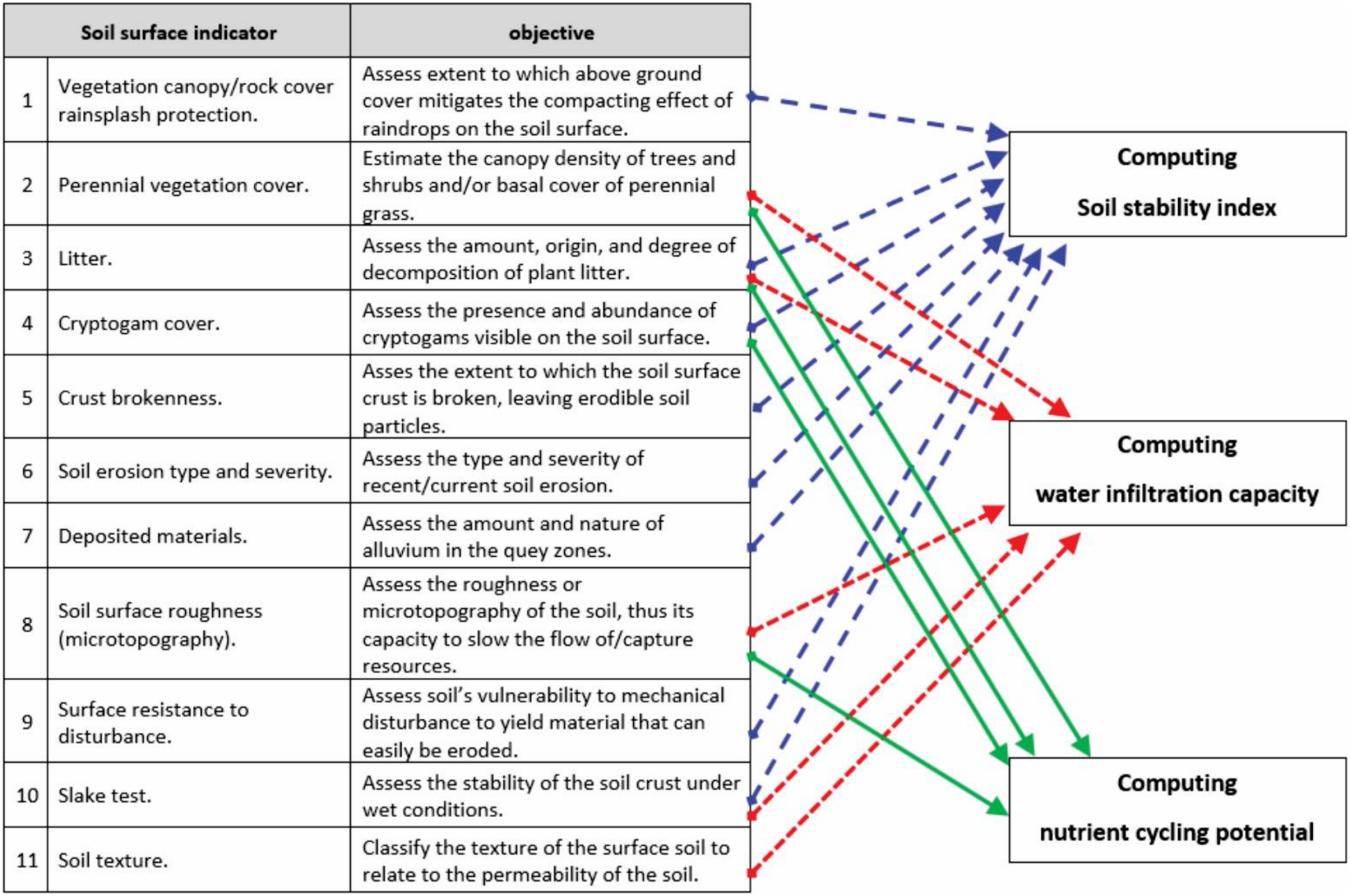
The combination of the 11 soil surface indicators to compute the three functionality indices namely soil stability, water infiltration capacity and nutrient cycling potential (adapted from Tongway & Hindley, 2004a). © Copyright CSIRO Australia. Computing soil stability index, water infiltration capacity and nutrient cycling potential.

The applied LFA protocol has two stages: the first stage is the landscape organisation measurements in which the patches and fetches are identified, and the dimensions of each patch are measured. The second stage is called soil surface condition assessment (SSA), in which 11 soil surface features are measured. These are soil cover (vegetation, bare ground, rock), litter cover, canopy cover, crust brokenness, cryptogam cover, erosion degree and type, accumulated materials, soil surface roughness, soil surface resistance to erosion, soil texture, and slake test (Tongway & Hindley, 2004b). From these 11 features, a further three landscape functionality indices were computed using the Excel spreadsheet (workbook) developed in 2003 by CSIRO Sustainable Ecosystems (based in Canberra, Australia). By integrating the 11 soil surface indicators data obtained from the field survey, the three indices were automatically computed by the programmed Excel spreadsheet. These computed indices are: (1) soil stability, a measure of how the soil withstands erosive forces or reforms after erosion, (2) water infiltration capacity, which indicates the extent to which rainfall infiltrates into the soil, and (3) nutrient cycling potential, which provides a measure of how efficiently organic material is cycled in the soil (Fig. 2) as described in Tongway & Hindley (2003).

The landscape organisation measurements and soil surface condition assessment exhibited the effect and interaction of all the environmental factors and ecological processes prevailing in the area which influence the ability of the landscape to capture and retain resources, *i.e*., the functional integrity (Kwok, Eldridge & Oliver, 2011). Low scores for these variables are equivalent for dysfunctional landscapes and high scores for fully functional landscapes (Rezaei, Arzani & Tongway, 2006; Kwok, Eldridge & Oliver, 2011).

All site features were recorded in the field, including coordinates, transect compass bearing, position in the landscape, slope, aspect, lithology, vegetation type, features of the soil surface, and digital photos were captured. LFA data were collected according to the protocols outlined in the technical manual by Tongway & Hindley (2004a). The data were collected directly along 100 m line transects, lying with the main direction of the runoff movement across the landscape, which is generally aligned with the main slope of the landscape.

Landscape organisation data were collected at the hillslope scale, which is a small unit representing catchment-scale runoff dynamics. The line transect was divided into areas where resources are captured (patches) and where resources are transported (fetches). The dimensions, location, and characteristics of each patch and fetch were measured according to the type and characteristics of the resource-regulating structures (Tongway & Hindley, 2004a, 2004b). From these measurements, the indicators of landscape organisation were computed as explained by various authors (Palmer et al., 2001; Randall, 2004; Tongway & Hindley, 2004a) and using the same Excel spreadsheet developed by CSIRO Sustainable Ecosystems.

The SSA data were collected at the patch-fetch scale. A set of query zones for SSA were initiated in each patch or fetch along the line transect. Query zones are chosen areas along the transect located within each patch and inter-patch type. The 11 indicators of soil condition were assessed in three to six replicates in each query zone type (Tongway & Hindley, 2004b; Van der Walt et al., 2012). Each SSA indicator ranging from 0 to 100 contributed to the total functionality of the landscape (Van der Walt et al., 2012) and was used to derive the three indices of soil surface condition (Fig. 2).

To assess the functional roles of vegetation, such as how the plants affect resource-regulating processes, the Point Centred Quarter method (PCQ), following Cottam & Curtis (1956) and Lindsey, Barton & Miles (1958), were used on the same 100 m LFA line transect. The sampling points for the vegetation survey were 5 m apart, and at each point, the interval distance to the nearest plant in each of the four quarters (sectors) around that point was measured, *i.e*., 20 points × 4 plants = 80 plants for each transect (McDonald et al., 1990). Additional attributes for each plant were recorded, including overall height, height to the canopy, width and breadth of the canopy, and canopy density (% of overall canopy space occupied by foliage and stems) (Tongway & Hindley, 2004b), and the dominant perennial plants were recorded at each site.

Due to the absence of any pristine site in the study area, the NDVI values of the park vegetation were calculated from Landsat 8 imagery acquired in September 2014. The NDVI values were used as a proxy of vegetation intensity, and in each of the vegetation types, the survey sites were categorised into three categories high, medium, and low vegetation intensity, according to the NDVI values in each vegetation type. These categories were different amongst the vegetation types due to the variation in environmental settings and vegetation characteristics across the diverse landscapes. These categories were used for comparing LFA variables between the different vegetation intensities within each of the three vegetation types.

All data were processed in a Microsoft Office Excel spreadsheet (Microsoft Corporation-2016). The total SSA functionality for each site was calculated from Eq. (1) (Tongway & Hindley, 2004a). Summation of the values calculated by Eq. (1) (total stability, total infiltration, and total nutrient cycling) was used in Eq. (2) to calculate the total SSA functionality for each vegetation type (Tongway & Hindley, 2004a; Van der Walt et al., 2012).

(1) $$\text{Total SSA for each site} = \sum \text{total stability; total infiltration; total nutrients}$$

(2) $$\eqalign{
  & {\rm{Total}}\;{\rm{SSA}}\;{\rm{for}}\;{\rm{each}}\;{\rm{vegetation}}\;{\rm{type}}  \cr 
  & {\rm{ = }}\sum ({\rm{total}}\;{\rm{stability}};\;{\rm{total}}\;{\rm{infiltration}};\;{\rm{total}}\;{\rm{nutrient}}){\rm{/N}}\;{\rm{sites}}\;{\rm{in}}\;{\rm{each}}\;{\rm{vegetation}}\;{\rm{type}} \cr} $$

SPSS Statistics version 22 (IBM, Armonk, NY, USA) was used for statistical and classification analysis. First, the data set was explored using descriptive statistics, and then normal distribution was tested using the Shapiro–Wilk W test (Shapiro, Wilk & Chen, 1968). ANOVA (one-way) followed by a Tukey HSD *post hoc* (Sokal & Rohlf, 1995) were used for examining any differences in patches and fetches characteristics, SSA variables, LFA indices, and vegetation characteristics amongst the different vegetation types and the differences amongst the various site categories (low, medium, and high vegetation intensity) within each of the vegetation types. In cases where the distribution of the data was non-normal, the non-parametric statistical test Kruskal–Wallis (one-way ANOVA on ranks) was used. The cover relationship between the different variables was explored using a bivariate correlation test. All statistical analyses were conducted at the 0.1%, 1%, and 5% significance levels.

The concept of sigmoid curves (S-shape) as described by Tongway & Hindley (2004a) and Read et al. (2016) was applied to illustrate the current position of LFA indices (soil stability, water infiltration, and nutrient cycling) for each vegetation type. As the survey sites were located carefully in each vegetation type to represent the range of cover in the study area, the highest and lowest values of LFA indices were considered as the upper and lower critical thresholds. The middle point between the upper and lower critical thresholds was regarded as the inflexion point representing the threshold of potential concern, where the resources change from loss from the landscape under the inflexion point to accumulation within the landscape above the inflexion point (Read et al., 2016).

Using Primer software (version 9), the survey sites were clustered using Bray–Curtis’ similarity after square root transformation, and functionality variables were ordinated using Principal Coordinates Analysis (PCo-A) to indicate any possible functionality gradient.

## Results

### Vegetation characteristics

The total perennial canopy volume for the entire study area was low, with a mean of 10.1 m^3^ per 100 m^-2^ (± SE 1.89) and showed a highly significant difference (*p* < 0.001) amongst the vegetation types. The TES had the highest mean values, and the TK had the lowest (Table 1). The site categories (high, medium, and low vegetation production) within each vegetation type showed highly significant differences (*p* < 0.001) in perennial canopy volume in all the vegetation types. The mean value of the number of plants for the study area was 11,435 plants per hectare (± SE 1,635.5), the highest mean value was in TES, and the lowest was in TK, with highly significant differences (*p* < 0.01) amongst the vegetation types (Table 1). TK showed significant differences (*p* = 0.004) amongst site categories in the number of plants per hectare, and no significant difference was detected within TWR and TES.

**Table 1:** Mean value (± SE) for the physical landscape heterogeneity parameters in the vegetation types: Tanqua Karoo (TK), Tanqua Wash Riviere (TWR), and Tanqua Escarpment Shrubland (TES) within the Tankwa Karoo National Park.

	TK	TWR	TES	*p*-value
Altitude (m a.s.l.)	411.60 (± 15.03)***	511.73 (± 20.82)	786.62 (± 47.36)	<0.001
Slope (%)	4.00 (± 1.33)***	2.73 (± 0.80)	15.77 (± 2.71)	<0.001
Total patches length m	7.85 (± 1.74)***	30.38 (± 2.78)	52.05 (± 3.21)	<0.001
Total patches width (m)	9.93 (± 2.32)***	43.95 (± 4.41)	78.33 (± 5.82)	<0.001
Total patches area (m^2^ per 1,000 m^−2^ of the ground)	4.20 (± 1.43)***	42.61 (± 9.08)**	70.92 (± 9.65)*	<0.001
No of (patches per 10 m^−1^)	3.58 (± 0.49) ***	6.09 (± 0.74)	10.07 (± 0.70)	<0.001
Patch area index (%)	0.003 (± 0.002)***	0.042 (± 0.009)**	0.075 (± 0.009)*	<0.001
Landscape organisation index	0.08 (± 0.02)***	0.30 (± 0.03)	0.55 (± 0.03)	<0.001
Mean fetch length (m)	3.07 (± 0.34)	1.64 (± 0.38)	0.49 (± 0.07)	<0.001
No of plants per hectare	8,407 (± 3,919)**	11,127 (± 1,811)	15,281 (± 1,935)	0.004
Mean interval distance between plants (m)	2.47 (± 0.43)	1.21 (± 0.16)	0.88 (± 0.07)	0.004
Soil stability (%)	51.43 (± 1.86)	55.03 (± 1.42)	57.62 (± 1.11)	0.025
Water infiltration capacity (%)	25.85 (± 1.16)	27.81 (± 1.54)*	29.75 (± 0.82)	0.104
Nutrient recycling potential (%)	14.47 (± 1.21)	17.29 (± 1.72)*	20.59 (± 1.12)	0.016
Perennial canopy volume (m^3^ per 100 m^−2^)	0.77 (± 0.23)***	6.78 (± 1.29)***	24.59 (± 3.50)***	<0.001

**Note:**

The last column demonstrates significant differences (*p*-value) amongst the vegetation types and the asterisk within a row illustrate the significant differences amongst high, medium, and low vegetation cover categories within each vegetation type, where: ***p < 0.001; **p < 0.01; and *p < 0.05.

**Figure 3:**
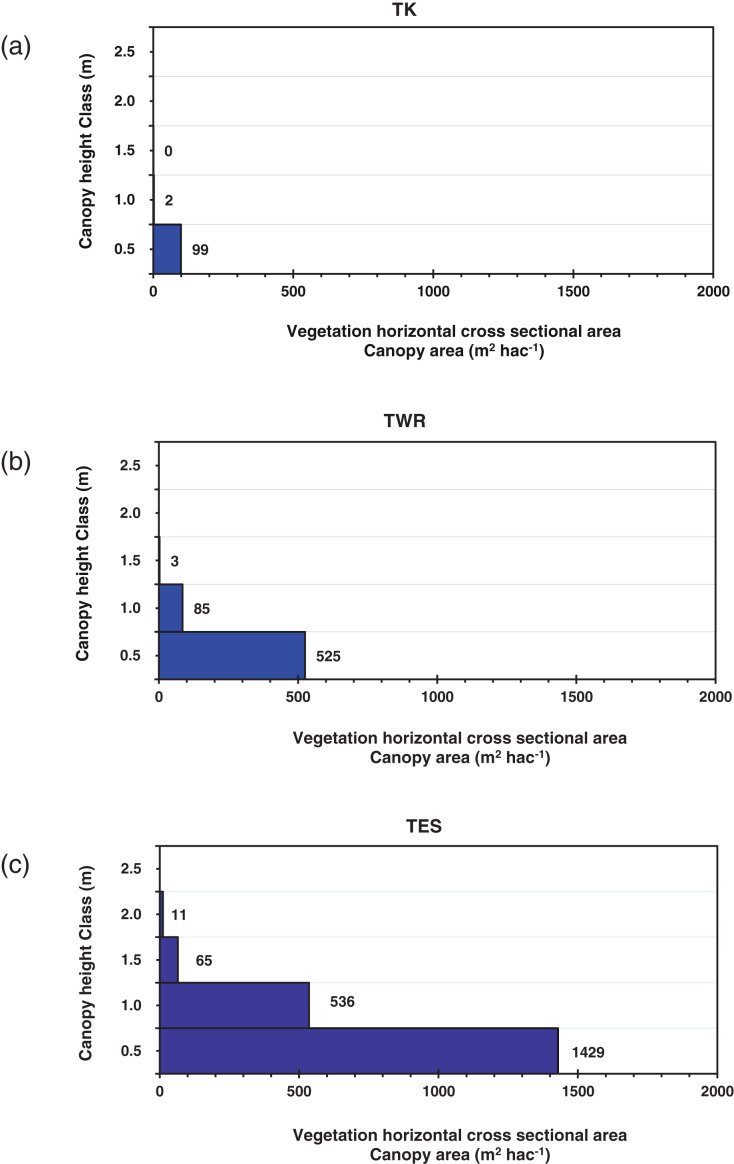
Mean horizontal canopy cross-section area (m^2^ hectare^−1^) and height distribution (m) of the plants in the three vegetation types. (A) Tanqua Karoo vegetation type (TK), (B) Tanqua Wash Riviere vegetation type (TWR), and (C) Tanqua Escarpment Shrubland (TES), divided into intervals of a vertical height of 0.5 m, showing the dominance of dwarf shrubs that have a height less than 0.5 m.

The mean interval distance between plants (distance/b/plants) overall for the area was 1.55 m (± SE 0.19), the highest mean value was in TK, and the lowest was in TES, with a highly significant difference (*p* < 0.01) amongst the vegetation types, while no significant difference was detected amongst site categories within all of the vegetation types. The bivariate correlation test showed highly significant positive correlations (*p* < 0.001) amongst altitude and slope *vs* perennial canopy volume and a highly significant negative correlation (*p* < 0.001) between altitude *vs* interval distance values between plants (Table 2). The horizontal canopy cross-sectional area (m^2^ per hectare) revealed that dwarf shrubs with a height of less than one meter dominate the area, while a small percentage of TES shrubs reached 2 m (Fig. 3).

**Table 2:** Correlation matrix depicting Pearson’s simple linear correlation coefficient (r) for landscape function parameters amongst all the investigated parameters overall for the vegetation types, Tanqua Karoo, Tanqua Wash Riviere, and Tanqua Escarpment Shrubland in the Tankwa Karoo National Park.

Variable	Altitude	Slope	Total patch length	Total patch width	Total patch area	Patches per 10 m	Patch area index	Landscape organisation index	Mean fetch length	Stability index	Infiltration index	Nutrient index	No. of plants per hectare	Mean interval between plants
Slope	0.754***													
Total patch length	0.798***	0.663***												
Total patch width	0.796***	0.739***	0.969***											
Total patch area	0.576***	0.656***	0.812***	0.837***										
Patches per 10 m	0.694***	0.419**	0.734***	0.695***	0.341*									
Patch area index	0.616***	0.656***	0.838***	0.855***	0.992***	0.398**								
Landscape organisation index	0.810***	0.667***	0.987***	0.964***	0.792***	0.774***	0.826***							
Mean fetch length	−0.642***	−0.434**	−0.768***	−0.738***	−0.452**	−0.862***	−0.480***	−0.777***						
Stability index	0.357*	0.316*	0.503***	0.509***	0.370*	0.334*	0.384*	0.512***	−0.227					
Infiltration index	0.316*	0.267	0.481***	0.493***	0.249	0.516***	0.253	0.481***	−0.515***	0.260				
Nutrient index	0.443**	0.438**	0.652***	0.664***	0.429**	0.540***	0.435**	0.645***	−0.518***	0.591***	0.805***			
No of plants per hectare	0.320*	0.042	0.269	0.239	0.033	0.653***	0.063	0.290	−0.592***	0.021	0.356*	0.249		
Mean interval between plants	−0.477***	−0.251	−0.581***	−0.549***	−0.336*	−0.659***	−0.357*	−0.586***	0.739***	0.059	−0.453**	−0.393**	−0.632***	
Perennial canopy Volume	0.781***	0.834***	0.834***	0.877***	0.768***	0.546***	0.776***	0.834***	−0.573***	0.483***	0.459**	0.576***	0.188	−0.395**

**Note:**

The asterisks illustrate the significant correlation (two-tailed) amongst the various parameters, where ***p < 0.001; **p < 0.01; and *p < 0.05.

The life-form of the plants in the study area consists mainly of ephemerals and annuals (therophytes) and dwarf and tall shrubs (chamaephytes) with some cryptophytes. The most common perennial plant species in the open plains (TK) were *Malephora crassa*, *Zygophyllum chrysopteron*, *Salsola aphylla*, *Cladoraphis spinosa*, *Drosanthemum lique*, *Augea capensis, Psilocaulon junceum*, and *Tripteris sinuata*. In the flood plains (TWR), the most common species were *Aridaria noctiflora*, *Salsola aphylla*, *Drosanthemum framesii*, *Malephora crassa*, *Ruschia centrocapsula*, *Zygophyllum chrysopteron*, *Tripteris sinuata*, *Galenia sarcophylla*, *Lampranthus otzenianus*, *Ruschia cradockensis*, *Pteronia glauca*, *Pteronia pallens*, *Drosanthemum lique*, *Galenia africana*, *Eriocephalus microphyllus*, *Euphorbia decussata*, and *Phyllobolus* spp. In the escarpment (TES), the most common species were *Pteronia pallens*, *Ruschia* spp., *Drosanthemum* spp., *Aridaria noctiflora*, *Euphorbia decussata*, *Ruschia centrocapsula*, *Justicia cuneata*, *Montinia caryophyllacea*, *Galenia africana*, *Euphorbia mauritanica*, *Drosanthemum lique*, *Tripteris sinuata*, *Pteronia glauca*, *Asparagus capensis*, *Pteronia incana*, *Merxmuellera stricta*, *Searsia undulata*, *Hermannia paucifolia*, and *Anisodontea triloba*.

The plant species on the escarpment consisted of a relatively higher percentage of succulent and woody dwarf shrubs and some tall shrubs, which generally have a larger size and a slower turnover dynamic (life span >10 years) compared to the plant species on the plains, which were dominated more by annuals, biennials, and dwarf shrubs (life span <10 years) (Stock, Dlamini & Cowling, 1999; Li et al., 2008).

### Landscape heterogeneity measurements (patches *vs* fetches)

Overall, the area’s landscapes consisted of an average of 29% patches *vs* 71% fetches. The TES had the highest mean values of patch dimensions and patch area, and the TK had the lowest values, with highly significant differences (*p* < 0.001) amongst the vegetation types. The TES had a mean patch area of 1.7 times higher than in the TWR and 16.9 times higher than in the TK (Table 1). The patch areas showed significant differences (*p* < 0.05) amongst site categories within all the vegetation types.

The TES had the highest mean number of patches per unit area, and the TK had the lowest value, with highly significant differences (*p* < 0.001) amongst the vegetation types. The TK revealed a highly significant difference (*p* < 0.001) amongst the site categories in the number of patches per unit area, while no significant differences were detected within the TWR and TES (Table 1).

The overall landscape organisation index value in the study area was 0.3 (± SE 0.03), with a highly significant difference (*p* < 0.001) amongst the vegetation types; in TES, it was 1.8 times higher than in the TWR and 6.9 times higher than in the TK. There was a highly significant difference (*p* < 0.001) amongst site categories in the landscape organisation index in the TK, while no significant differences were detected within the TWR and TES. The opposite was true for mean fetch length values, where TK had the highest value, and TES had the lowest value, with a highly significant difference (*p* < 0.001) amongst the vegetation types, and no significant differences amongst site categories within all the vegetation types (Table 1).

The altitude and slope showed a highly significant positive correlation (*p* < 0.01) *vs* all the landscape heterogeneity parameters, except for the mean fetch length which had a highly significant negative correlation (*p* < 0.01) (Table 2).

### Soil surface assessment

The SSA showed low values of soil stability, water infiltration capacity, and nutrient cycling potential where the mean values for the study area overall were 54.6% (± SE 0.94), 27.7% (± SE 0.74), and 17.3% (± SE 0.88) respectively; the TES had the highest values, and TK had the lowest values (Table 1). There were significant differences (*p* < 0.05) amongst vegetation types in soil stability and nutrient cycling potential, and no significant difference was detected for water infiltration capacity.

**Figure 4:**
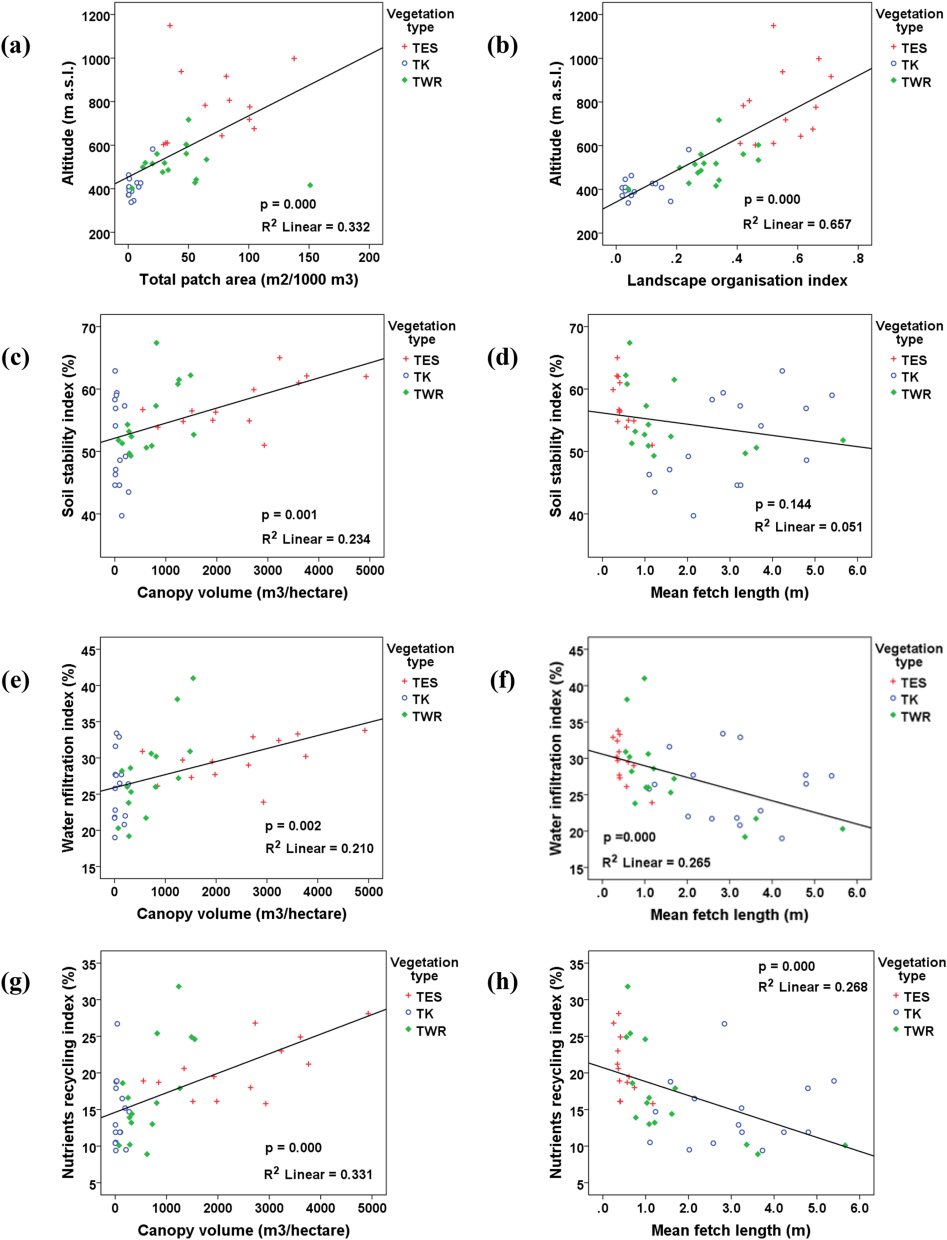
Biplots (A–H) illustrating the correlation between different landscape parameters in the three vegetation types. Tanqua Karoo (TK), Tanqua Wash Riviere (TWR), and Tanqua Escarpment Shrubland (TES) within the Tankwa Karoo National Park.

Within vegetation types (vegetation categories), there were no significant differences in soil stability, water infiltration, and nutrient cycling in the TK and the TES. The TWR showed significant differences (*p* < 0.05) in water infiltration capacity and nutrient cycling potential (Table 1). Soil stability, water infiltration, and nutrient cycling were significantly positively correlated (*p* < 0.05) to altitude, patch area, landscape organisation index, and perennial canopy volume. Water infiltration and nutrient cycling were significantly negatively correlated (*p* < 0.05) to interval distance between plants (Table 2 and Fig. 4).

**Figure 5:**
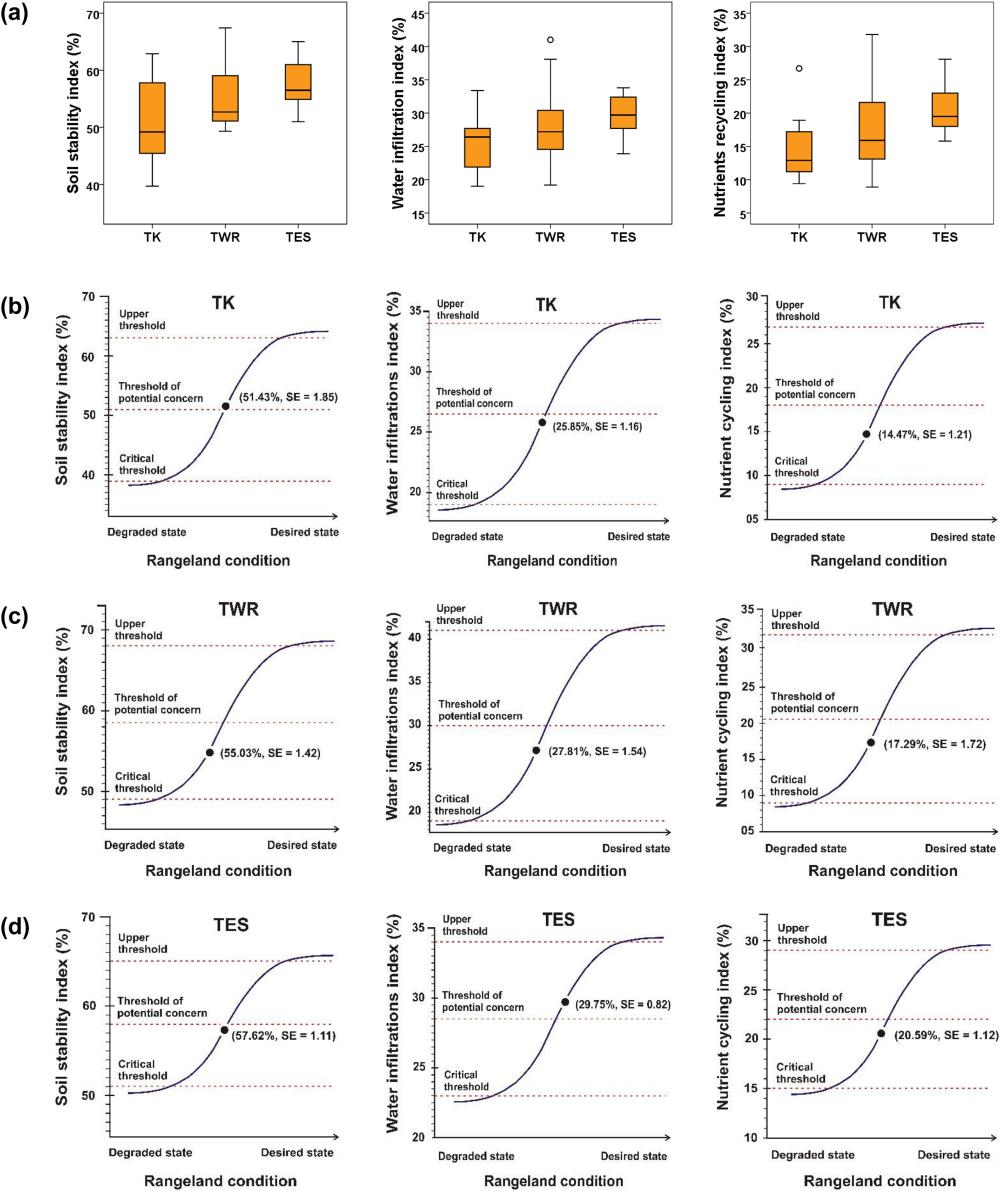
Graphs for landscape function indices (soil stability, water infiltration, and nutrient recycling) in the different vegetation types: Tanqua Karoo (TK), Tanqua Wash Riviere (TWR), and Tanqua Escarpment Shrubland (TES) within the Tankwa Karoo National Park. The first row (A) depicts box-and-whisker plots showing the median (midline box) and the first and third quartiles of the data. Bars show the 95% confidence interval level; the data not included between the whiskers represent outliers. The second to fourth rows (B–D) depict sigmoid curves (S-shape); the position of landscape function indices on the curve represents mean (± SE) of LFA indices for the different vegetation types in its current state, the points of maximum curvature represent the upper and critical thresholds, and the inflection point represents the threshold of potential concern.

The sigmoid curves (S-shape) showed that the mean values for all the indices in the three vegetation types were low and below the threshold of potential concern, except for the soil stability index for TK, which is slightly above the threshold line, and the water infiltration index for the TES (Fig. 5). The plains, notably the TWR, were more dysfunctional than the escarpment.

**Figure 6:**
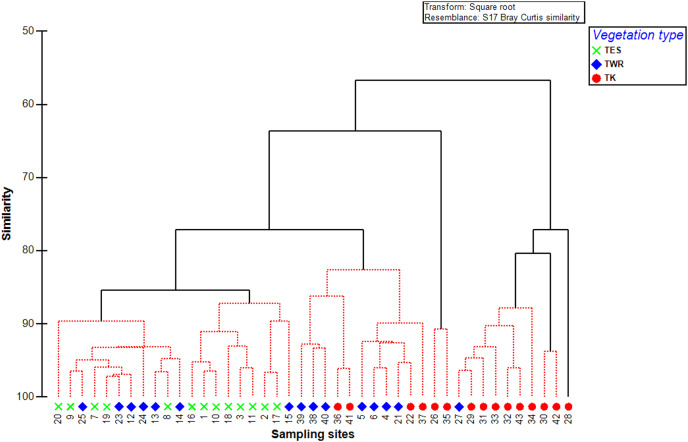
Clustering dendrogram of Bray–Curtis similarity after square root transformation for the 43 sampling sites in the different vegetation types. Tanqua Karoo (TK), Tanqua Wash Riviere (TWR), and Tanqua Escarpment Shrubland (TES) within the Tankwa Karoo National Park.

The cluster analysis of the survey sites demonstrated a clear separation between the TES and TK sites and the TWR sites distributed in the middle (Fig. 6). The Bray–Curtis similarity result at 60% similarity categorised the survey sites into two main groups, at 65% similarity into three sub-groups, and at 80% similarity into five sub-groups.

**Figure 7:**
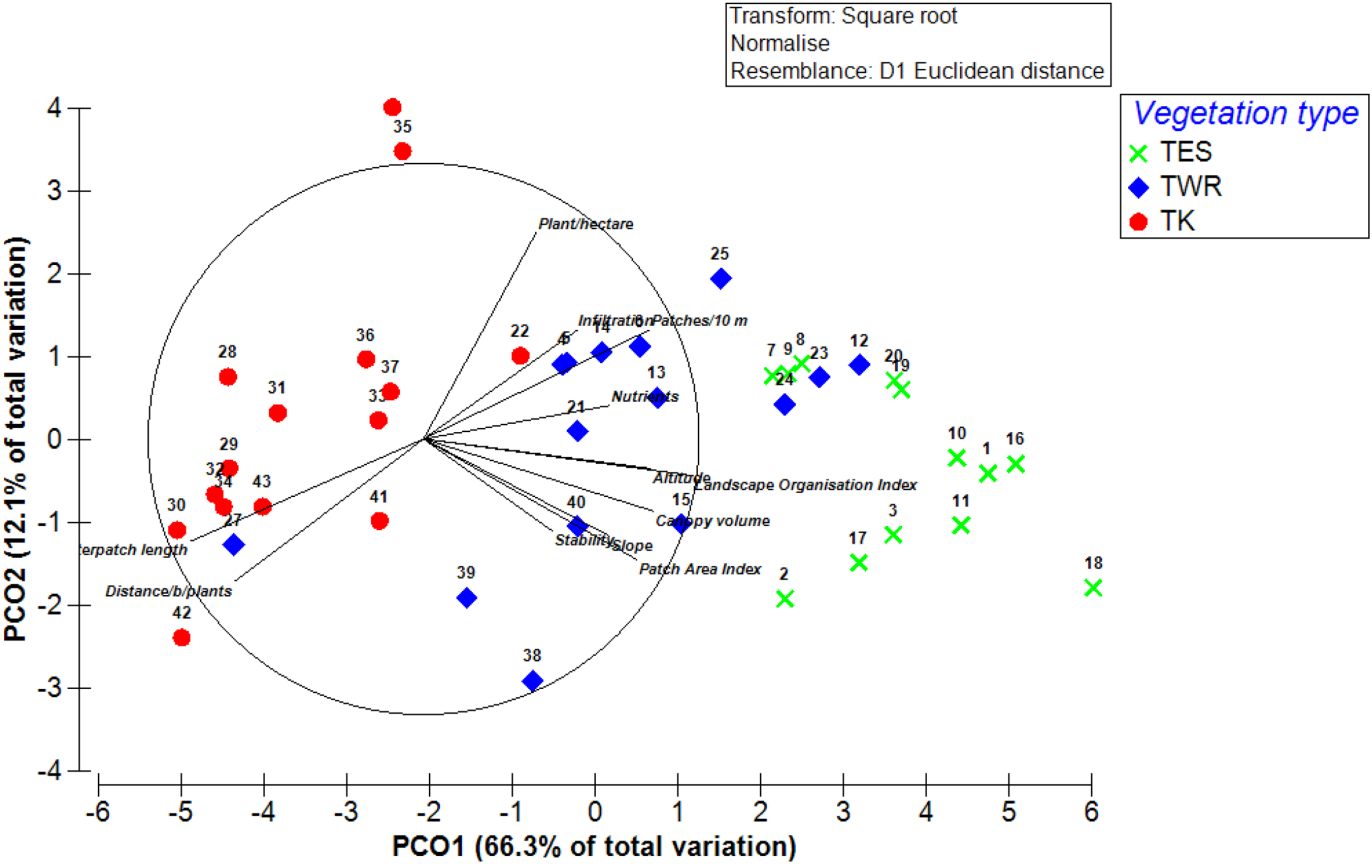
The ordination diagram represents the first two axes of a Principal Coordinates Analysis (PCo-A) for the distribution of the sampling sites (from 1 to 43) based on the different variable results after square root transformation. The first two axes explain 78.4% of the variation between the survey sites in the different vegetation types: Tanqua Karoo (TK), Tanqua Wash Riviere (TWR), and Tanqua Escarpment Shrubland (TES) within the Tankwa Karoo National Park.

Also, the ordination using Principal Coordinates Analysis (PCo-A) showed that the first and second axes captured 78.4% of the total variation among the survey sites. The eigenvalue along the first axis was 445.52 and captured 66.3% of the total variation. Along the second axis, the eigenvalue was 81.298 and captured 12.1% of the total variation (Fig. 7). The first axis was positively related to the altitude, slope, canopy volume, patch area index, landscape organisation index, water infiltration index, and nutrient cycling index, and negatively related to interpatch length and patches number per unit length. The second PC axis was positively related to the number of plants per unit area and negatively related to the mean interval between plants and soil stability index.

## Discussion

### Vegetation characteristics

Vegetation characteristics are essential indicators of landscape function, *i.e*., the balance between resource loss and retention (Bastin et al., 2002; Mahmoud et al., 2014). Thus, it comprises a foundational component in functional dynamics across the landscape. For optimal function, a landscape must have a high perennial canopy volume and a high diversity of plant life-forms (Kearns & Barnett, 1999). Canopy volume increases the accumulation of the organic matter on the soil and reduces the rain-splash effect, thus reducing erosion, and high plant life-form diversity providing different ecosystem services.

The vegetation analysis in the study area showed low canopy volume and low diversity of plant life-forms dominated by dwarf shrubs and annual herbs, which all point to a degraded landscape. The sparse distribution of the plant, high fetch percentage, and low life-form diversity all indicate a landscape with low functionality. This suggests the landscape cannot retain and utilise the resources effectively and is, thus, vulnerable to loss of ecosystem services such as fodder supply, carbon sequestration potential, and others.

The low proportion of perennials also reduces landscape function indices, which in this study varied across the study area in relation to the conditions of the vegetation types. This finding supports the work done by Tongway & Hindley (2004b) and Rezaei, Arzani & Tongway (2006), who stated that vegetation characteristics strongly influence landscape function indices.

In the study area, the escarpment had a substantially higher canopy volume and plant density and smaller fetches when compared to the plains; these were the most critical parameters that yielded significantly higher landscape function in the escarpment. This difference in landscape function amongst vegetation types indicates high variability in environmental conditions in the park, which could be attributed to the differences in rainfall between the escarpment and plains and historical utilisation mainly by livestock. As this protected area expanded over the years, several farms were acquired at different times, and those farmers adopted different stocking rates and livestock management strategies.

The significant positive role of higher altitude on most vegetation characteristics and patchiness was expected due to the role of altitude in changing microclimate and the rockier slopes are known to limit accessibility and utilisation of resources by local people and wild and domestic animals. This resulted in significant differences in vegetation structure and shrub size between the higher-lying areas on the escarpment and the lower-lying areas on the plains. However, the same positive significant impact of the slope was unexpected and inconsistent with the findings of Bastin et al. (2002) and Rezaei, Arzani & Tongway (2006), who illustrate that the slope has a negative impact on vegetation characteristics and patchiness. This could be attributed to the fact that the steepness of the slope (degrees) for the survey sites on the escarpment is not very high (<40%). The vegetation survey in this study as part of the LFA illustrated the different vegetation conditions due to the different environmental settings and historical utilization intensity.

### Landscape heterogeneity (patches *vs* fetches)

In contrast to the fetch areas, the patch areas play a significant role in landscape functionality *via* capturing and utilising organic matter, nutrients, water, and sediments (Ludwig et al., 2002). Therefore, they have deemed indicators of the extent to which a landscape is functional or degraded (Bastin et al., 2002; Van der Walt et al., 2012). To a certain degree, rangeland production in arid environments becomes relatively concentrated in patches when natural resources have a heterogeneous distribution (Noy-Meir, 1973). This is because vegetated patches generally score the highest functionality indices, while denuded fetches donate their natural resources to the nearby patches. However, a continuous decrease in the landscape organisation index and long fetches enhance runoff and accordingly increase erosion potential (Holm, Loneragan & Adams, 2002a; Kakembo, 2009), resulting in a leaky and degraded landscape.

On the escarpment, vegetation patches tend to coalesce; hence it had the largest patch size, patch number, patch area index, landscape organisation index, and the shortest fetch interval distances. This means that the landscapes on the escarpment have a higher ability to retain resources, and therefore are more functional and have a higher potential for improvement when compared to the plains. This finding is very influential in assessing the different levels of rangeland degradation amongst the various vegetation types, thus determining the appropriate rehabilitation programme for each one and anticipating the outcome even before the rehabilitation processes are applied.

The significant differences in vegetated patchiness features amongst site categories (within the examined vegetation types) revealed a high heterogeneity within these vegetation types. The most increased heterogeneity was in the TK, which could be another indicator of disturbance within this vegetation type, while the TES had the least heterogeneity, which reveals more stability within this vegetation type. Schlesinger et al. (1990) suggested that any process that leads to an intense heterogeneity of soil resources in space and time is likely to exhibit more significant levels of degradation in arid regions.

The differences in the examined vegetation parameters between the escarpment and plains sites were sufficiently pronounced to be separated in ordination space. The fact that TWR sites were located in the middle of the ordination diagram indicates that it can be reviewed as a transitional state between the TK and TES, which illustrates that it has not been much degraded compared to TK and more than TES. However, a few sites in the TWR showed a close similarity to sites in the TK.

The correlation and ordination analysis elucidated a substantial influence of altitude and slope on patch/fetch number, size, and distribution. These two physical aspects appear to be the main factors that control vegetation cover and landscape functionality, where the density and size of resource-accumulating patches decline from the higher-lying areas on the escarpment towards the lower-lying areas in the plains. This general declining trend was expected since altitude usually ameliorates the microclimate on higher-lying areas, resulting in denser vegetation cover. Many previous studies in the Succulent Karoo Biome and other arid areas elsewhere also highlighted the role of altitudinal gradients on microclimate and vegetation variation across landscapes (Kraaij & Milton, 2006; Perroni, Montaña & García, 2010; De Boever et al., 2016).

### Soil surface assessment

The soil stability index contributed the most to landscape functionality in all the examined vegetation types, and the nutrient cycling index contributed the least. The low contribution of nutrient cycling indicates low litter and organic matter accumulation on the soil surface due to the low vegetation cover. As a result of low cryptogram cover, soil stability is mainly governed by vegetation, coarse fragments on the soil surface, and physical crust, which differ amongst and within the various vegetation types.

Amongst the examined vegetation types, the TES had the highest scores of soil stability, water infiltration, and nutrient cycling indices and thus were deemed more able to retain resources. This is likely due to the naturally engineered slope character and altitude effect on microclimate, which is mirrored as higher patchiness measurements. However, the landscapes on the plains are believed to be leakier due to the lower canopy volume and patch sizes as the aridity increases as one moves westwards, away from the escarpment.

Although the results showed highly significant differences amongst vegetation types in most of the landscape heterogeneity indices, this is not reflected with the same degree of differences at the three LFA composite indices of functionality. This disagrees with the observations in other areas in South Africa and other places worldwide (Tongway & Ludwig, 1997a; Van der Walt et al., 2012). This could be a unique feature for the Tanqua Karoo region, where the relatively rapid replacement of the plants (short-lived shrubs and annual herbs) constituting the vegetated patches does not allow the vegetation to ameliorate the soil characteristics significantly, such as modifying soil texture, increasing litter cover, increasing organic matter content, and increasing cryptogam cover. These are the major factors influencing soil stability, water infiltration, and nutrient cycling (Tongway & Ludwig, 1997b).

The only significant difference among vegetation cover categories within the vegetation types was in water infiltration and nutrient cycling in the TWR. These indices are influenced mainly by accumulated organic matter on the ground surface and soil texture. The homogeneity in most of LFA indices within the vegetation types suggests that the altitude and location in the landscape are the most influential factors on landscape functionality, where each vegetation type lies in a specific range of elevation and has its local environmental conditions, which do not create any variation within these vegetation types.

Based on the strength of the correlation, the most important factors that influence the variation in landscapes functionality in the study area are vegetation characteristics, altitude, and slope degree, respectively. This finding is in line with the outcomes of Rezaei, Arzani & Tongway (2006) in an arid area in Iran and Munro et al. (2012) in an arid area in Australia. These factors control vegetation characteristics and patchiness, which contributes significantly to the buffering capacity of the landscape, *i.e*., the ability of a landscape to sustain ecological processes and maintain the functionality of an ecosystem when subject to a disturbing influence (Haagner, 2008).

Even though most of the park’s area has been under conservation for three decades, the sigmoidal curve illuminated that the landscape is still generally dysfunctional for all the examined vegetation types, and significant improvement in landscape functionality will most likely not occur in the near term as a direct result of existing low patchiness, higher fetch space, low LFA indices, and the low annual rainfall of the region. This suggests that the ecosystem is unlikely to improve without an active intervention to elevate it from a dysfunctional state below the threshold of potential concern to a functional state above the threshold. This active intervention should differ in the various vegetation types based on the degradation level of each one of them.

## Conclusions

Although this study showed a general dysfunctional state of the TKNP, which necessitates an active intervention, there were different levels of dysfunctionality amongst the examined vegetation types in the study area. The escarpment seems to have a higher ability to capture, retain, and utilize natural resources than the plains; thus, it has a higher potential for improvement.

The significant differences amongst site categories within vegetation types were more apparent on the plains than the escarpment, which is most probably because of the differences in rangeland condition due to the harsh environmental factors prevailing in the area and historical overgrazing. This implies that the LFA approach works well and would inform management and track rehabilitation over time in arid rangelands. Rangeland recovery would occur at a much faster rate on the escarpment than on the plains, and this requires more active interventions on the plains. However, active rehabilitation should occur within each vegetation type based on the fetches *vs* patches state. Rehabilitation teams and park management should heed these results because widespread active restoration should not increase patches *vs* fetches beyond the recommended or appropriate conditions as shown by LFA across and within vegetation types. A consequence would be that the dynamics of the rehabilitated vegetation would not be consistent with adjacent unimpacted areas.

Although LFA failed in detecting variation in functional landscape attributes in the Namaqualand in a previous study (Petersen et al., 2004), this study illustrated that LFA could be used effectively to assess the ecosystem state in the arid Tanqua Karoo region, and it provided additional insight into the rangeland condition and showed the poor rangeland condition of the study area in terms of a dysfunction landscape. Park management should employ the LFA approach to track rehabilitation areas against the natural trajectories of change. This study illustrated that the concept of landscape functionality and its application techniques are applicable in arid rangeland areas and could play a vital role in rangeland management, particularly in the assessment of degradation and monitoring rehabilitation processes. In the case of TKNP, this technique showed the different levels of degradation in the various vegetation types within the park, so different rehabilitation programmes are required in each vegetation type. Based on other interventions concurrently happening in two arid protected areas, the only limitation when applying LFA approach for management of rehabilitation or restoration sites is that it should be supported by suitable benchmarks or the Before-After-Control-Impact-Paired (BACIP) study/project design. LFA approach was able to provide insights into rangeland conditions in the Tanqua Region.

## Supplemental Information

10.7717/peerj.13305/supp-1 Supplemental Information 1 Raw Data.

10.7717/peerj.13305/supp-2 Supplemental Information 2 LFA vegetation assessment - Heterogeneity.

10.7717/peerj.13305/supp-3 Supplemental Information 3 Soil surface indices.

## Additional Information and Declarations

### Field Study Permissions

The following information was supplied relating to field study approvals (*i.e*., approving body and any reference numbers): South Africa National Parks Research Review Committee.

### Data Availability

The following information was supplied regarding data availability: The raw data is available in the Supplemental File.
